# A stream processing abstraction framework

**DOI:** 10.3389/fdata.2023.1227156

**Published:** 2023-10-25

**Authors:** Ilaria Bartolini, Marco Patella

**Affiliations:** Department of Computer Science and Engineering (DISI), Alma Mater Studiorum, University of Bologna, Bologna, Italy

**Keywords:** stream processing, real-time analysis, Big Data, multimedia data streams, software framework

## Abstract

Real-time analysis of large multimedia streams is nowadays made efficient by the existence of several Big Data streaming platforms, like Apache Flink and Samza. However, the use of such platforms is difficult due to the fact that facilities they offer are often too raw to be effectively exploited by analysts. We describe the evolution of RAM3S, a software infrastructure for the integration of Big Data stream processing platforms, to SPAF, an abstraction framework able to provide programmers with a simple but powerful API to ease the development of stream processing applications. By using SPAF, the programmer can easily implement real-time complex analyses of massive streams on top of a distributed computing infrastructure, able to manage the volume and velocity of Big Data streams, thus effectively transforming data into value.

## 1. Introduction

Starting from early 90's, multimedia (MM) data have been employed in a wide range of applications. The widespread accessibility of such data is made possible by the availability of inexpensive production (cameras, sensors, etc.) and storage technologies. Moreover, MM data frequently arrives in streams, or successions of the same sort of MM objects, which are received from a producer.

Significant value can be mined from MM streams, but there are also significant demands placed on computational capacity and analytical ability (Tang et al., 2016). Real-time analysis, in particular, necessitates the processing of data streams at high throughput and low latency in order to take advantage of data freshness to act and make judgments rapidly.

A number of Big Data platforms exist (Zaharia et al., 2010; Alexandrov et al., 2014; Noghabi et al., 2017) that provide services for the management and analysis of massive amounts of information, enabling evidence-based decision making across a wide range of human activities. However, the usage of such platforms is complicated for analysts, because their primary attention is on issues of fault-tolerance, synchronization, increased parallelism, and so forth, rather than offering programmers an intuitive interface.

In this paper, we show how RAM3S (Bartolini and Patella, 2018, 2019, 2021)—a framework that we developed to integrate Big Data management platforms (RAM3S stands for Real-time Analysis of Massive MultiMedia Streams)—has evolved int SPAF, a Stream Processing Abstracting Framework. The use of SPAF makes much easier, for researchers, to implement real-time complex analyses of massive MM streams exploiting a distributed computing environment, without specific knowledge of the underlying stream processing distributed infrastructure.

After introducing the running example that will be exploited throughout the paper to illustrate RAM3S/SPAF concepts (Section 2), we will briefly describe the scenario that originally led us to introduce RAM3S and present its current structure (Section 3). The key concepts in SPAF are illustrated in Section 4, while Section 5 provides details on how the stream processing model considered by SPAF can be extended to deal with Direct Acyclic Graph topologies. We will then proceed to analyze the time/space overhead of the SPAF framework (Section 6) and to discuss some of the developments we are working on to be included in the next SPAF release (Section 7). Finally, Section 8 concludes, by also providing a multi-variable analysis of SPAF.

## 2. Running example: face recognition

In May 2023, Italy's Interior Minister Matteo Piantedosi, interviewed by a national newspaper, claimed that the Italian government was “considering an extensive video surveillance system with facial recognition capabilities” to be deployed in “in highly frequented places” such as stations, hospitals, and commercial areas, in order to contrast the (at the moment) recent rise of crimes and acts of violence.^1^ Besides the obvious concerns about the privacy of citizens and its trade-off with their security/safety, the proposal was also interesting for its impact on the technological infrastructure of smart cities. Indeed, in this application, several cameras are disseminated in the territory (airports, metro stations, public buildings, and so on), producing videos. In order to provide automatic facial recognition, each video has to be first streamed as a sequence of frames and each incoming frame analyzed to:

(1) Verify if it contains a face;
(2) Compare the (possibly) discovered face against a number of “known” faces, to retrieve the known face most similar to the input face;
(3) The face is regarded as correctly recognized if there is a high enough similarity score between the newly discovered face and its most similar known face; otherwise, it is regarded as an unknown face.

**Figure 1:**
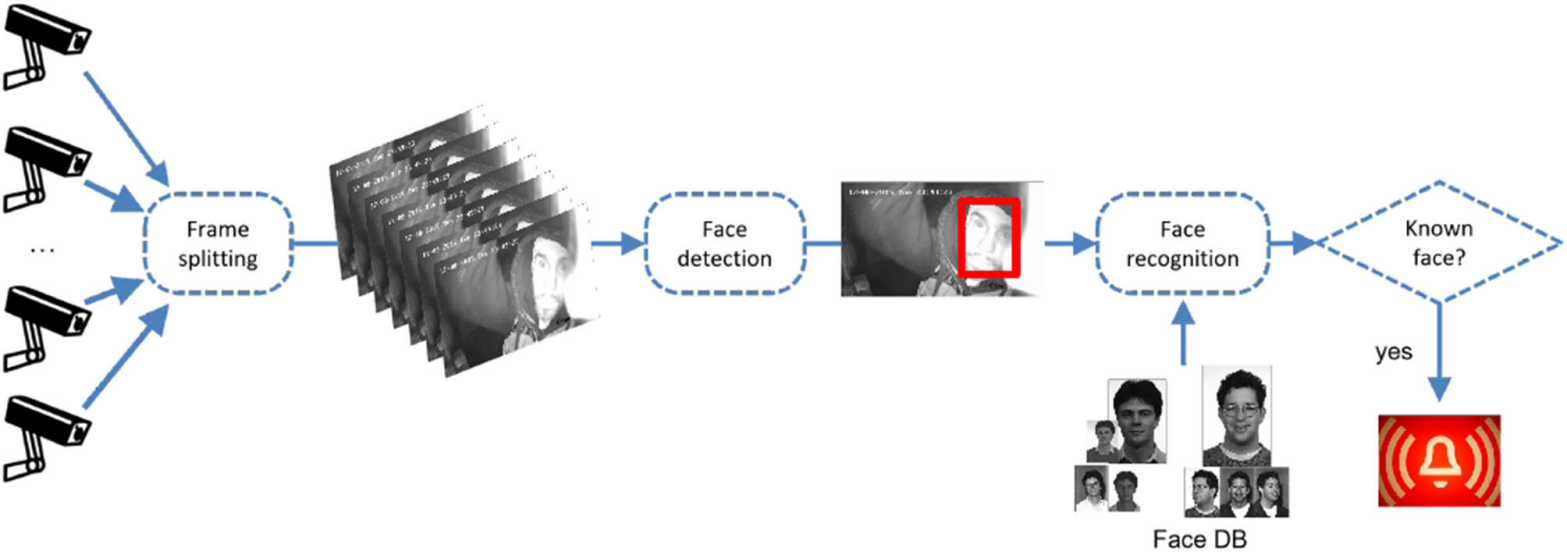
Face recognition use case.

Since performing all above tasks within the camera itself would require expensive hardware (as well as a way to upload “known” faces), solutions based on edge computing can be hardly applied. Rather, a data infrastructure has to be devised to (1) send videos from cameras to a data processing platform and (2) to analyze such videos on the platform as previously illustrated (see Figure 1). The amount of data coming from several thousands of cameras around Italy would clearly prevent the use of a centralized platform. On the contrary, distributed computing can be of help, because every single frame might be analyzed independently of the others, so that processing of incoming videos could be performed in parallel.

Automatic facial identification in videos was the first application of RAM3S that we created (Bartolini and Patella, 2018).^2^ For this use case, we exploited the well-known Viola–Jones algorithm (Viola and Jones, 2001) for face detection, while comparisons of “faces” was performed using a technique based on principal component analysis using eigenfaces (Turk and Pentland, 1991). Clearly, one could consider using different techniques for face detection/recognition, with different precision and time complexity: The use of any particular algorithm is independent on how the computation is distributed on the processing platform, which is oblivious of the tasks it performs.

**Figure 2:**
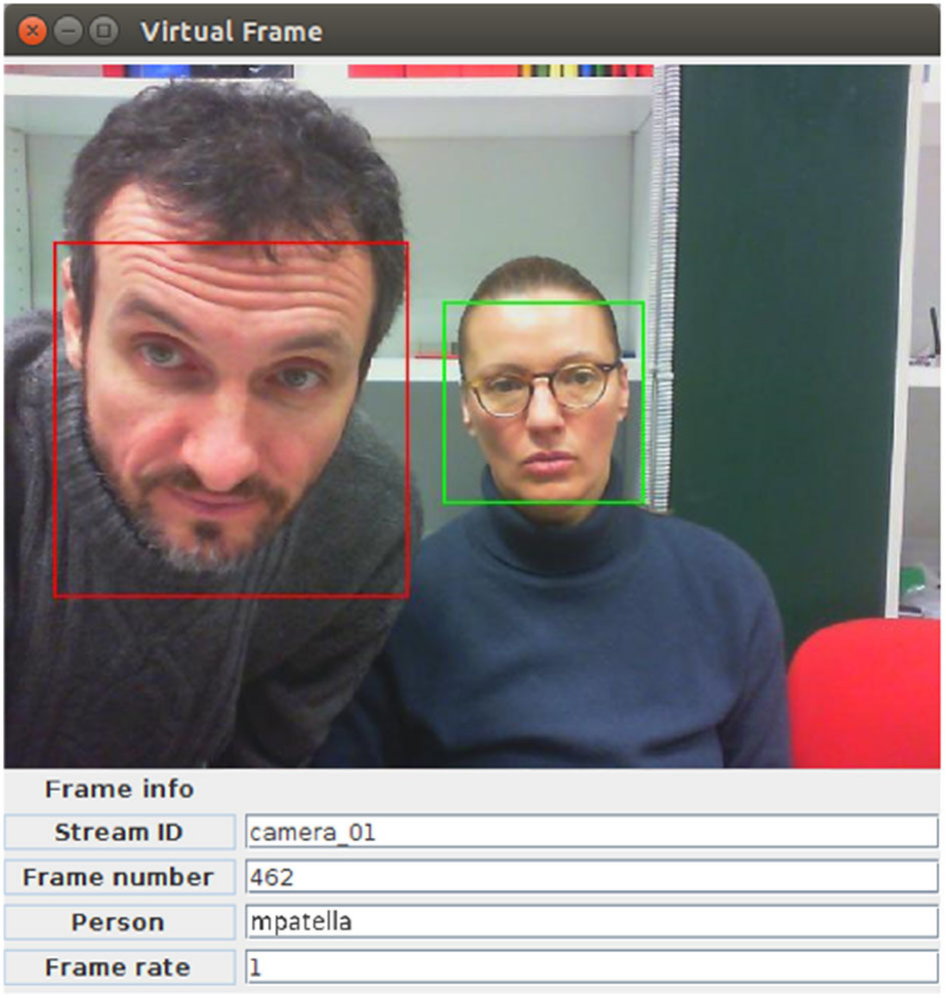
The GUI of the suspect face detection RAM3S prototype: the left person is correctly recognized (“mpatella,” included in the face DB), the right person is correctly unrecognized (not included in the face DB).

For the purpose of suspect identification, every time a detected face is sufficiently similar to one of the faces in the knowledge base, the prototype we implemented to illustrate the use of RAM3S raises an alarm. This is also reflected in the prototype GUI (see Figure 2), which outputs incoming images with a frame enclosing each detected face with color red (if recognized) or green (not recognized).

We will use the face recognition use case presented here in the remainder of the paper to instantiate many RAM3S (and SPAF) components. In this way, we will provide concrete form to abstract concepts, so as to improve their comprehensibility to the reader.

## 3. Introducing RAM3S

Our experience with Big Data streaming analysis platforms derives from a private-financed project focused on the analysis of multimedia streams for security purposes. The private company already had an experience in Big Data *batch processing*, through the long term use of tools such as Hadoop (http://hadoop.apache.org) and Spark (http://spark.apache.org). However, it was clear that the use of such technologies was inappropriate for a number of security tasks, such as face detection for the automatic identification of “suspect” people (Turk and Pentland, 1991), recognition of suspicious behavior from videos (Mu et al., 2016), human actions (Zhang et al., 2016) or gesture (Roh and Lee, 2015), audio events (Łopatka et al., 2016), and so on. Indeed, in such *online* applications, data have to be analyzed as soon as these are available, so as to exploit their “freshness.” The storing of incoming data is, thus, usually unnecessary and the efficiency of the system depends on the amount of data processed, keeping low latency, at the second, or even millisecond, level.

To deal with this (at the time) novel *stream processing* paradigm other Big Data platforms were introduced, among which Storm (http://storm.apache.org), Flink (http://flink.apache.org), and the streaming version of Spark. Abstracting by specificities of each Stream Processing Engine (SPE) [see Zaharia et al. (2010) and Alexandrov et al. (2014) for details on Spark and Flink, respectively], the following common characteristics can be discovered [see also Bartolini and Patella (2018) for a more detailed comparison of the SPEs originally considered in RAM3S]:

- Some nodes in the architecture are in charge of receiving the input data stream, thus containing the data acquisition logic (these are called Receivers in Spark, Spouts in Storm, and Producers in Flink).
- Other nodes perform the actual data processing (these are called Drivers in Spark, Bolts in Storm, and Task Nodes in Flink).
- Finally, data sources and data processing nodes are connected to realize the data processing architecture, which takes the form of a Directed Acyclic Graph, where arcs represent the data flow between nodes (in Storm, such architecture is termed Topology).

We were, therefore, challenged to implement a number of security-related use-cases on top of such SPEs, with the goal of comparing them by way of several performance KPI, such as throughput, scalability, latency, etc. This required to re-implement every multimedia stream analysis application on top of each SPE, leading to huge code replication and other inefficiencies. For this, we considered realizing a middleware software framework to allow users to:

(1) avoid having to deal with details of each specific SPE (such as how fault-tolerance is achieved, how stream data are stored, etc.), and
(2) easily extend already available (centralized) software to a *scaled out* solution.

This way, we strove to bridge the technological gap between facilities provided by SPEs and advanced applications (for which transition to a distributed computing scenario might not be straightforward). RAM3S represents, as far as we know, the only attempt to provide a general perspective on the analysis of massive multimedia streams, providing the programmer with an abstract view hiding details and complexity of distributed computing.

### 3.1. RAM3S: *almost* a framework

Our original goal for RAM3S was to create a *framework*, according to the definition provided in Gamma et al. (1995):

“*A* ***framework*** *is a set of cooperating classes that make up a reusable design for a specific class of software…You customize a framework to a particular application by creating application-specific subclasses of abstract classes from the framework.”*

A framework, therefore, determines the overall architecture of an application and focuses on the *reusability* of a solution architecture, exposing to application programmers only parameters necessary to realize the desired behavior, as enacted through the internal mechanisms of the framework itself. Applications developed on a framework have therefore some degrees of freedom, that must be however provided by the framework a-priori. For this latter reason, it is of paramount importance that the framework is also characterized by *extensibility*, since it is practically impossible to predict all the needs of concrete applications in advance; this way, the framework will be able to better withstand the test of time, since it can eventually evolve through possible future integrations.

The current version of RAM3S allows to experiment with the various SPEs (namely, Spark Streaming, Storm, Flink, and Samza) by providing a separate “generic” application for each of them. Each of such applications allows the execution of the specific example on the respective SPE. RAM3S interposes an abstraction layer based on interfaces between the generic and the example applications. Such interfaces model aspects of both data streaming and data processing:

- The Receiver interface represents the external system from which the application receives data. The receive method accommodates the logic of receiving the single processable object from the external system.
- The Analyzer interface represents the container of all the processing logic of the application: its analyze methods takes a MM object as input, generating a single object as result.
- Finally, the ApplicationFactory interface is responsible for representing the application as a unit; in essence, it serves as a collector for the Analyzer implementation and for the Receiver implementation, by instantiating the concrete Analyzer and Receiver type classes defined in the application context.

**Figure 3:**
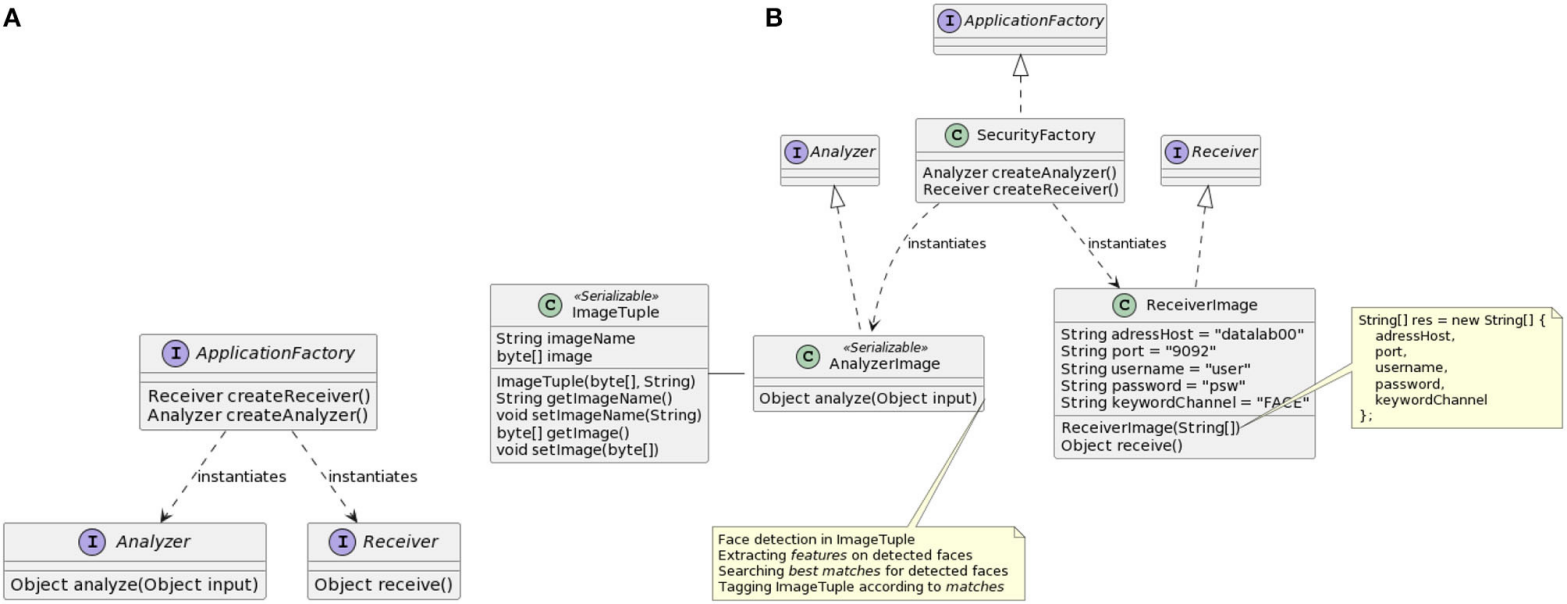
RAM3S programming interface **(A)** and its instantiation for the face recognition application **(B)**.

Figure 3 illustrates (Figure 3A) the relationships between RAM3S interfaces and (Figure 3B) how these are implemented for the face detection example.

**Figure 4:**
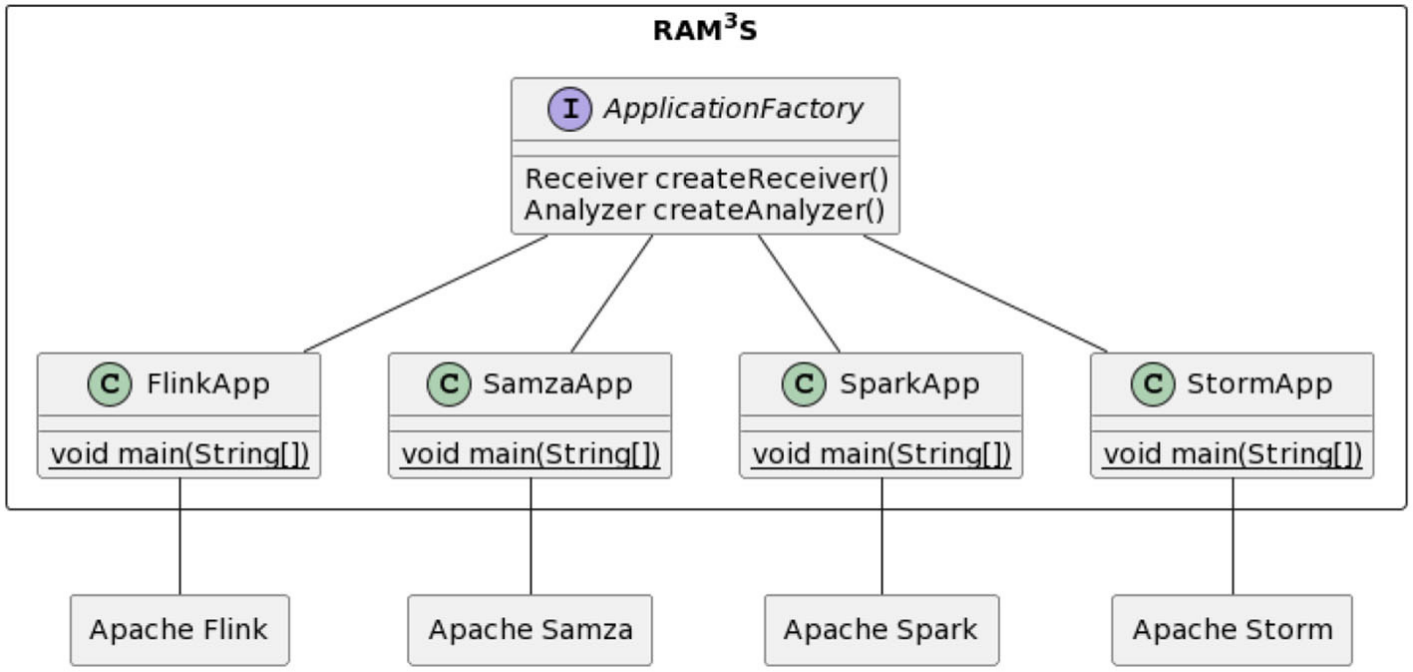
RAM3S generic applications for all included stream processing engines.

Let us now consider how RAM3S interfaces are used by generic applications. The purpose of a generic application is to map the application (defined in terms of the above interfaces) to the relevant SPE, therefore executing its logic on the underlying runtime framework. The class diagram in Figure 4 contextualizes the RAM3S generic applications with respect to the various stream processing engines considered in RAM3S.

**Figure 5:**
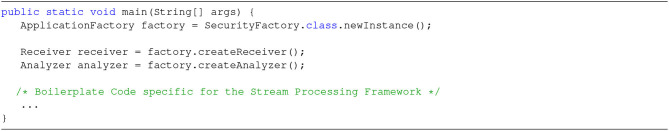
Code of RAM3S for the face recognition application.

Generic application code is always completely specified within the main method and has a recurring structure, presented in Figure 5.

The concrete factory is used to create the Receiver and Analyzer; then, such objects have to be translated into objects and procedures specific of the underlying SPE, as exposed by its APIs; the purpose of this step is to concretely establish the connection to the specific data processing infrastructure and to implement the application logic. Clearly, this is so-called boilerplate code, peculiar to the underlying SPE, that has to be repeated, almost verbatim, for each specific application. Moreover, code specific for a single application is repeated (again, almost verbatim) over the main method of any used SPE.

**Figure 6:**
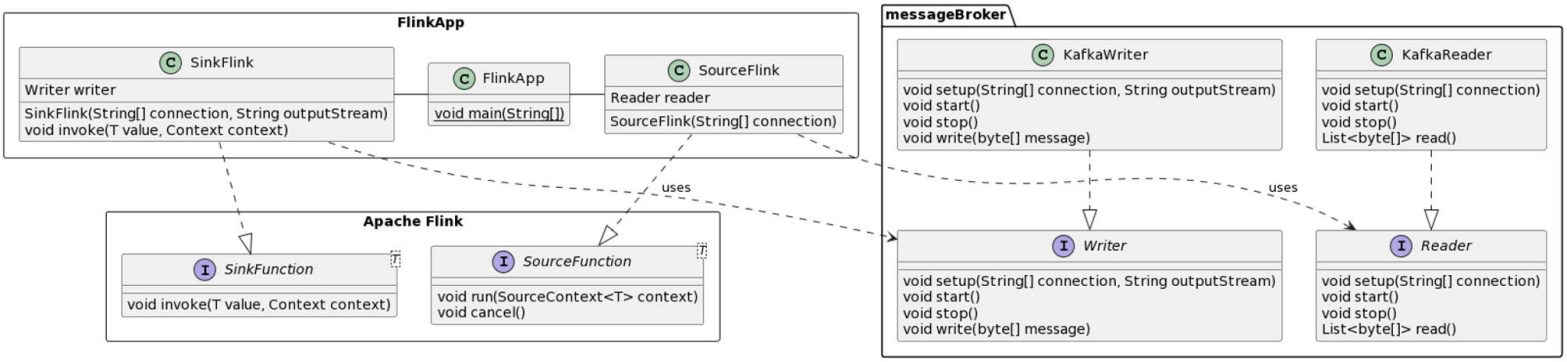
RAM3S support for message brokers.

A final component of RAM3S is the one used to support different message brokers (Bartolini and Patella, 2021). The abstraction layer devoted to message brokers consists of the messageBroker package shown in Figure 6. This package includes interfaces for the abstract representation of “readers” and “writers” (Reader and Writer interfaces) and concrete classes for the implementation of interfaces for a specific message broker (the figure reports only those for Apache Kafka, i.e., KafkaReader and KafkaWriter). For each stream processing engine (Figure 6 depicts the example of Flink), an additional layer is needed to map the above Reader and Writer concrete classes into, respectively, the source and destination of data processed by the application.

From the analysis we have presented, we can conclude that, at present, RAM3S allows different stream processing applications to be executed in a facilitated manner, but is not yet able to allow the definition of a new application without having to write part of the code of RAM3S itself. This drawback places RAM3S in the role of a “quasi-framework.” On the other hand, the message broker support is independent of the application code (thus satisfying the reusability requirement) and makes it possible to decouple the implementation of the read and write “adapters” of a certain message broker from the underlying stream processing engine (thus also offering extensibility). In the next section, we will introduce SPAF, whose main goal is to enable the use of RAM3S according to the original intent, namely to facilitate the creation of new stream processing applications, while striving for both reusability and extensibility.

## 4. From RAM3S to SPAF

Before describing the concepts that have been used to define our Stream Processing Abstraction Framework, SPAF, it is useful to recollect the original requirements for RAM3S:

**Facilitate the creation of stream processing applications:** the SPAF API should explicitly expose the pivotal concepts of stream processing and, more importantly, allow the application to be defined by writing code as close as possible to a description in natural language. Checking type-safety at compile time would be also helpful.
**Framework independence:** here, the target user is not the programmer of applications, rather the developer who wants to extend SPAF to work with a different SPE. Such programming interface is called Service Provider Interface (SPI) and is a programming pattern supported natively by Java.
**Connector independence:** it should be possible to integrate, in a pluggable manner, new connector providers (e.g., message queues, file systems, databases), again exposing a SPI to be implemented by developers. SPAF will therefore expose a dual abstraction layer: one for SPEs and one for input/output supports. Connector abstraction actually concerns the application programmer as well, since the SPAF API should relieve the programmer of the implementation details regarding the use of each connector's libraries and allow her to specify sources and destinations in a declarative manner.

### 4.1. Simplifying assumptions

In the first version of SPAF, a number of simplifying assumptions has been introduced to ease implementation of the previously described requisites:

(1) Connectors will be limited to message queues, as provided by well-known message brokers such as Apache Kafka and RabbitMQ.
(2) Streams will be composed exclusively by key-value pairs.
(3) No support will be provided for storing intermediate computation results, i.e., stream processing will be stateless.
(4) The processors operating transformations on streams will accept a single input stream and a single output stream; essentially, it will be possible to define only “linear” topologies.
(5) It will be possible to specify only the logical topology (definition of the transformation process from input to output) and not the physical topology (definition of how various computational elements of the logical topology—sources, transformation operators, destinations—are to be distributed on the physical nodes).

Assumption 1 can be easily overcome by providing tools (e.g., a command-line script) able of reading data to be processed from the desired source type (e.g., an input file) and publishing them to an appropriate input queue; likewise, it is possible to implement scripts able of consuming messages from the output queue and store them on the desired destination (e.g., a database). As for Assumption 3, our choice was to favor generality and simplicity over completeness. Indeed, not all SPEs might support stateful computation (for example, Storm does not) and those that support it introduce several other concepts that would complicate the SPAF model of stream processing. Although this version of SPAF does not include formal, integrated support for storing (globally or locally) intermediate results of individual transformation nodes, nothing prevents the programmer to implement access to storage resources external to the framework (e.g., files, DBs, object caches) in the transformation logic defined in the processing nodes, i.e., the programmer is responsible for implementing support for stateful computation. In this way, complex algorithms for analyzing multiple objects, like interval joins (Piatov et al., 2021), can be mapped to single processing nodes that are part of a more complex topology, performing other analyses (like image processing and/or event detection) on data streams (Persia et al., 2017). While a possible solution to overcome Assumption 4 will be described in Section 5, Assumption 5 can be partially solved by connecting multiple independent RAM3S applications by way of appropriate connectors; in this way, each logical topology would be mapped to a physical topology (defined automatically by, and peculiar of, the SPE chosen for each RAM3S application). Since this so-called *super-*topology can assume the form of a Direct Acyclic Graph (DAG), this can also be viewed as a solution to Assumption 4.

### 4.2. SPAF architecture

**Figure 7:**
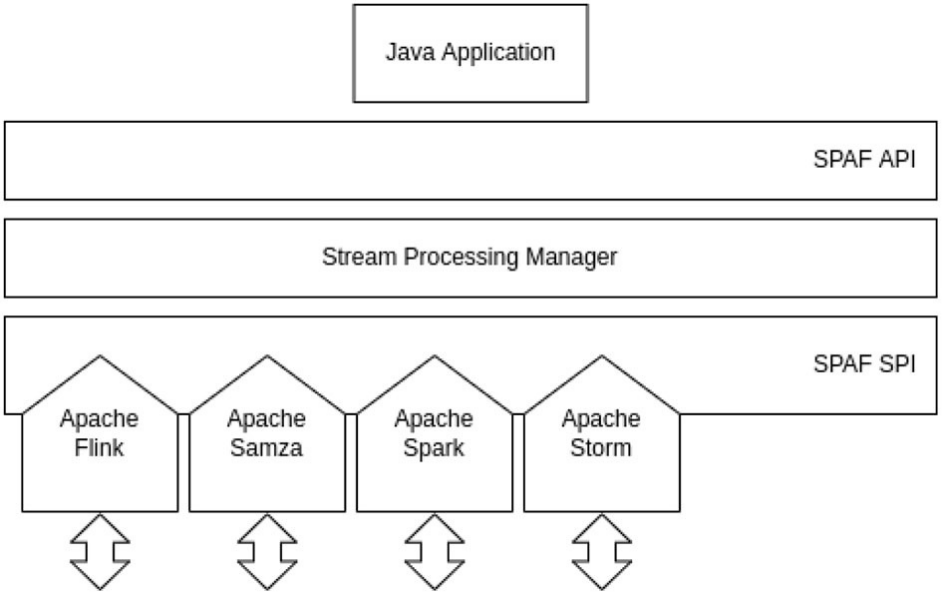
SPAF architecture.

Figure 7 shows the general architecture of SPAF. The application programmers will use the *user-facing* API to implement their stream processing application using Java code. Developers wanting to extend SPAF to include new SPEs (or connectors) will use the *provider-facing* SPI to write the *glue code* that allows bridging SPAF concepts to those peculiar to the SPE.

### 4.3. SPAF concepts

**Figure 8:**
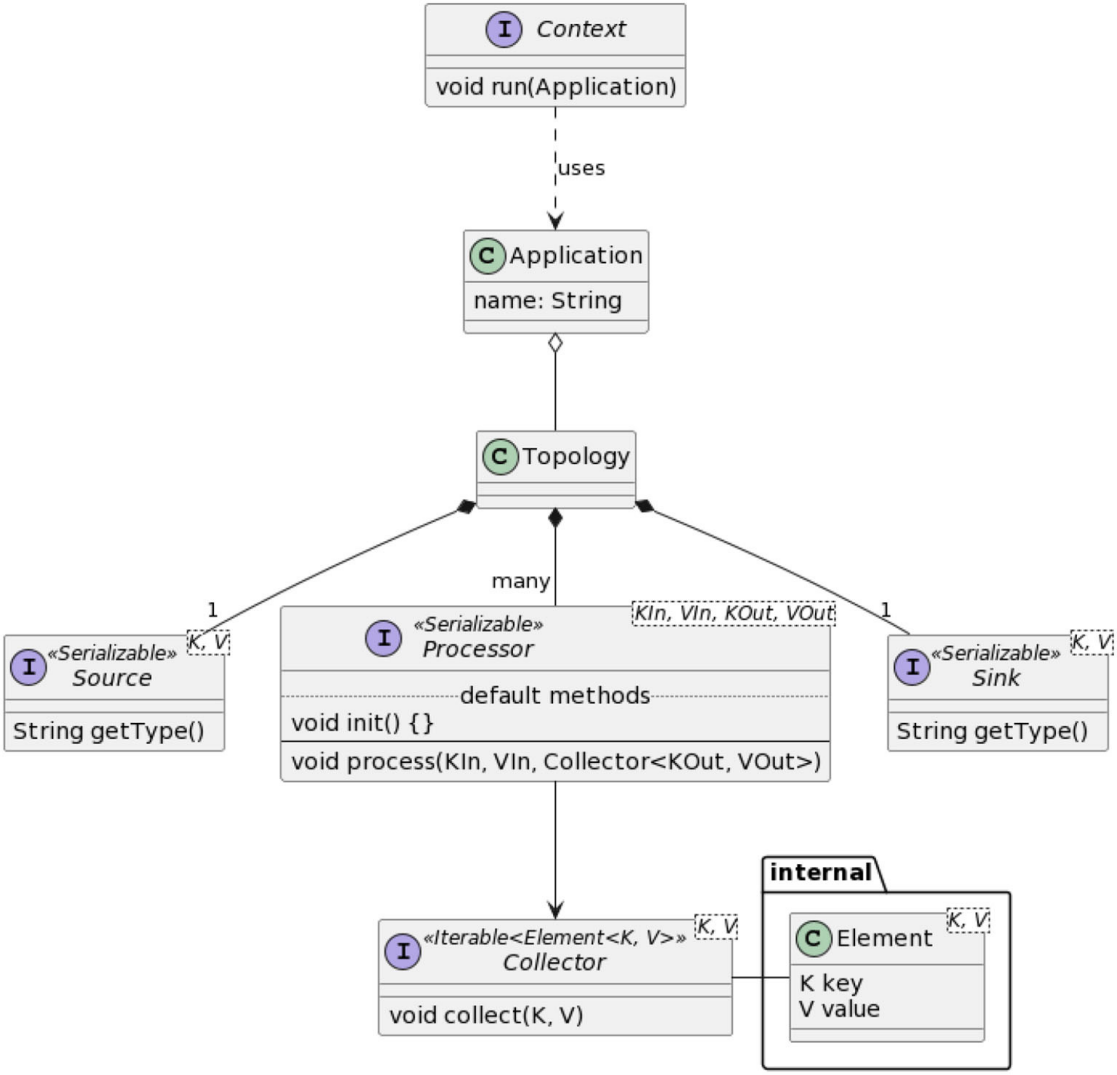
Main SPAF classes and interfaces.

The first step in the definition of SPAF was to provide a general model of the stream processing problem, thus defining a set of entities that are common to all SPEs (see Figure 8).

The Context entity represents the actual execution environment in which the abstractly defined application will be implemented. This therefore acts as a “bridge” between the abstract SPAF world and the concrete world made available by SPEs. The run method of the Context class is the “entry point” of the stream processing application, just like the main method of a Java class. Through the run method, we submit as a parameter the application, defined in terms the SPAF API to the underlying SPE; the SPE will then “interpret” the application, “translating” it into an equivalent representation using its specific concepts. Implementation of the Context is thus totally peculiar to each SPE; for this, the class Config allows the programmer to configure the execution environment in an abstract way (on the API side) and to implement such configurations in a provider-specific way (on the SPI side).

A Topology defines the computational logic of a stream processing application, that is, how the input data are transformed into the output data. As said, in the first version of SPAF only linear topologies are supported. We conceived the framework to accept specifications of the logic of Processor nodes also via lambda-functions and present a *fluent* API to compose the topology. To achieve this, we exploited a “creational-type” design pattern called *Builder*, allowing complex objects (like a topology) to be constructed step by step; this could also re-used in future versions of SPAF, where it may be convenient to change the way the topology is represented (e.g., to encompass DAG topologies).

The concept of Application basically coincides with the one of Topology, essentially adding descriptive information only. Conceptually, however, a stream processing application could define more than one topology: this is why the two entities are separated, although, in this first version of SPAF, there is a 1-1 relationship between Application and Topology.

Source and Sink clearly denote the source and destination, respectively, for data in a stream processing application.

A Processor represents a node in the Topology, implementing a processing step that is used to transform data, thus realizing the actual data processing logic. Processors can be thought as “black boxes” with a single input and a single output stream, where data transformation can be defined arbitrarily through the process method. The additional init method can be defined in those cases needing a one-off initialization of the Processor.

Finally, the Element entity represents the only data type that can be streamed in a SPAF Topology. The Element class is somewhat hidden from the application programmer, while its use appears evident in the SPI layer, for both SPEs and connectors.

We finally provide in Table 1 a mapping between SPAF concepts and those encountered in the considered SPEs.

**Table 1:** Mapping between SPAF and SPE concepts.

**SPAF**		**Apache Samza**	**Apache Flink**	**Apache Storm**	**Apache Spark**
Context	*Local*	LocalApplicationRunner	LocalStreamEnvironment	LocalCluster	JavaStreamingContext
		*Remote*	RemoteApplicationRunner	RemoteStreamEnvironment	StoreSubmitter	JavaStreamingContext
Application		TaskApplicationDescriptor	StreamExecutionEnvironment	LocalCluster StoreSubmitter	JavaStreamingContext
Topology	*Logical*		StreamGraph	StormTopology	DStreamGraph
		*Physical*	StreamTask	JobGraph	Task	
Element		Object	Tuple0, …, 25 < T> DataStream < T>	Tuple	DStream < T>
Source		InputDescriptor	SourceFunction < T>	Spout	InputDStream
Processor		StreamTask::process()	ProcessFunction:: processElement()	Bolt	DStream methods
Sink		OutputDescriptor	SinkFunction < T>	Bolt	DStream::foreachRDD()

### 4.4. Developing an application using SPAF

To create an application in SPAF, it is necessary to follow some steps, mostly independent of the specific streaming application logic to be created. In the following, we will exemplify the creation of an application for the “face recognition in videos” example introduced in Section 2. What we want to prove with this “experiment” is that SPAF is indeed able to satisfy the requirements listed in Section 4, i.e., framework and connector independence and ease of creation of the application. As it will be clear, the application-specific code can be easily distinguished from the generic SPAF-based application code.

The code needed for the complete specification of a SPAF application has actually a dual nature:

- Some *declarative* code, included in a configuration file, needed to specify the customization of SPAF entities, like Context, Source, or Sink.
- Some *procedural* code, used to instantiate SPAF classes and to specify the actual stream processing application logic; this is included in the main method of the application and, again, is mainly boilerplate code.

**Figure 9:**
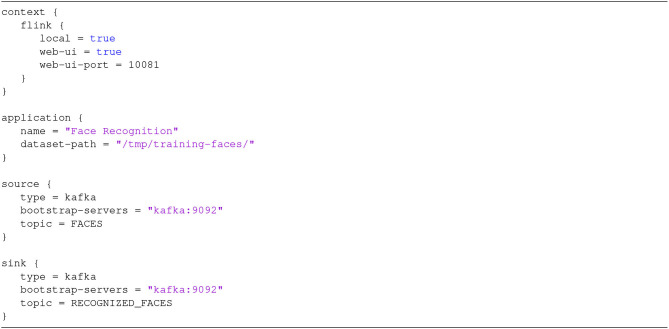
Configuration file for the SPAF face recognition application.

For the specific use case, a possible config file is shown in Figure 9, where one can recognize the specification of the context (using Flink as SPE) of the application, and of the input and output connectors (using Kafka).

**Figure 10:**
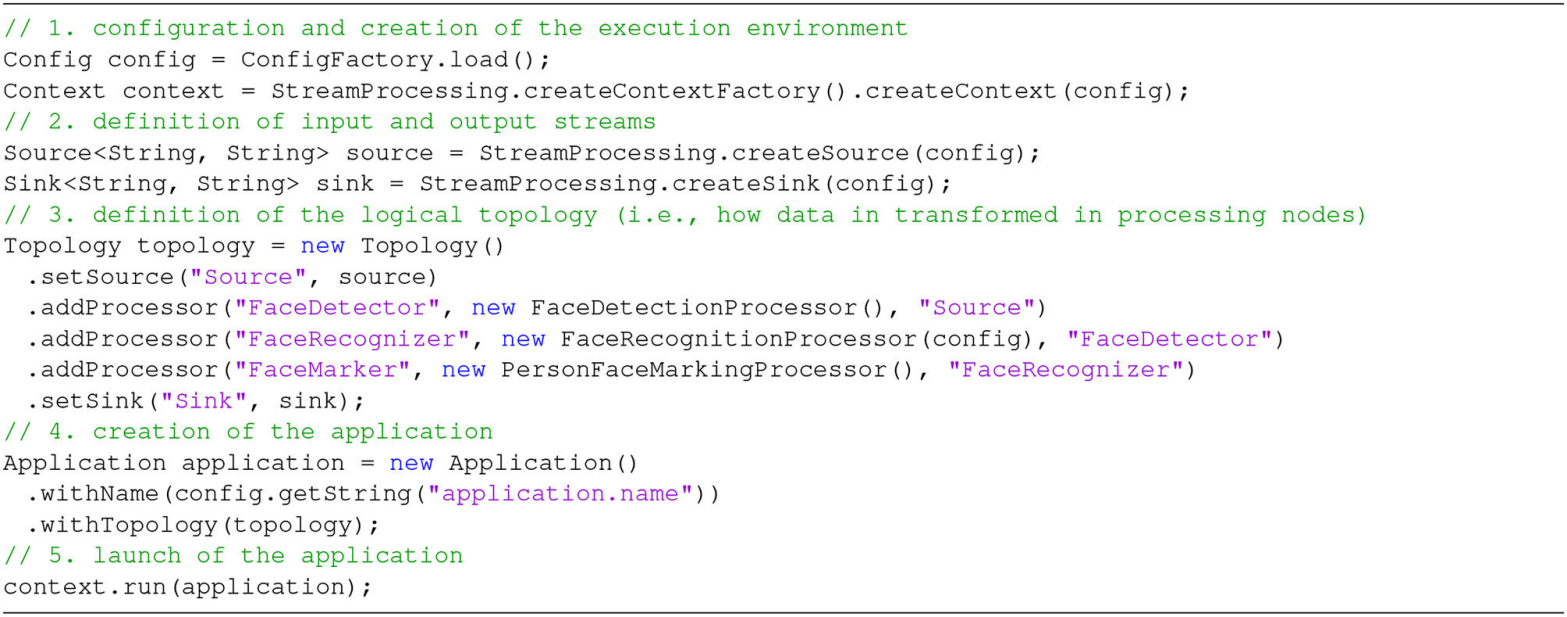
main method for the SPAF face recognition application.

Finally, the FaceRecognition application includes a main method with the code included in Figure 10.

**Figure 11:**
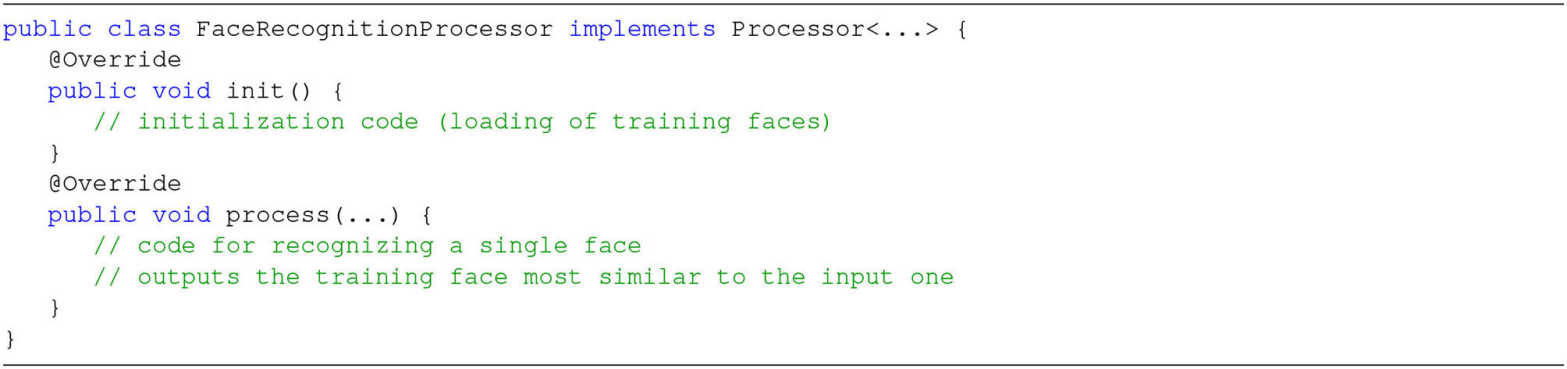
Implementation of a Processor in SPAF.

The goals of independence on frameworks and connectors are demonstrated by the fact that it is quite easy to change the configuration file to encompass different SPEs (in the context section) or connectors (source and sink sections). As to the creation of stream processing applications, it is clear that most of the procedural code is indeed boilerplate, i.e., repeated with no variation across different applications. The only specific part is at Step 3, where we specify that this application is composed of three main phases: (1) detection of faces in each image, (2) recognition of detected faces, and (3) (possible) marking of recognized faces (note that these correspond exactly with the three steps illustrated in Section 2). Obviously, the programmer should also write the code for each individual Processor (Figure 11 shows the example for the FaceRecognitionProcessor), but this is absolutely independent of the underlying stream processing infrastructure (following the original goal of RAM3S). Step 3 also demonstrates the use of the Builder pattern, where each Processor refers to its predecessor through its id.

A dimension of fundamental importance, which should be taken into account when choosing any development tool, is the so-called “learning curve”, which relates the level of knowledge and the time invested in learning a new thing. SPAF plays the role of the “guide” that takes the programmer through the discovery of stream processing concepts, providing a logical path that facilitates their understanding and thus making the learning curve of stream processing less daunting. SPAF is therefore able to make life easier for the inexperienced programmer who must venture into the world of stream processing for the first time, and make that journey less treacherous (like Virgil, accompanying Dante through the “hell” of stream processing).

## 5. Discussion

In this section, we want to highlight two interesting concepts, both related to the constructions of DAG-shaped topologies, namely the SPAF representation of topologies and the so-called *super-topologies*.

**Figure 12:**
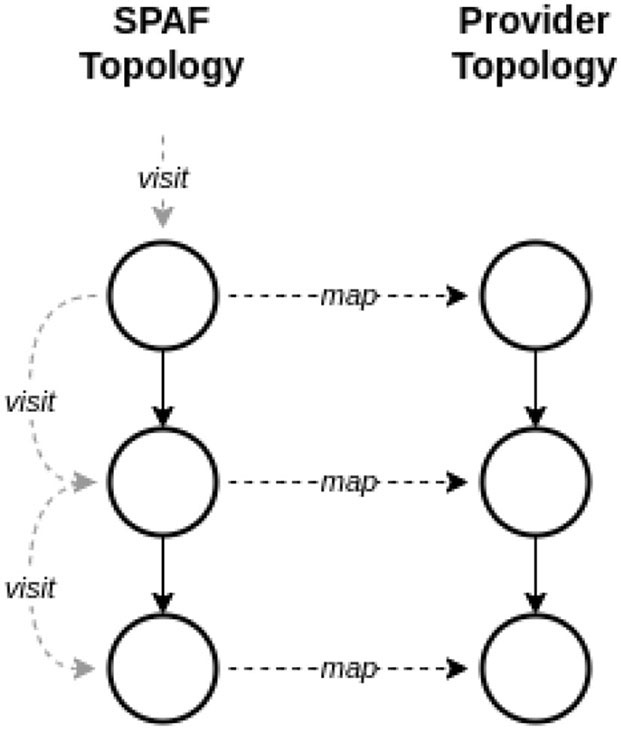
Mapping topologies.

When considering the representation of topologies in SPAF, we should remind that the key operation for a topology is how the SPAF stream processing layer (see Figure 7) is able to map the Topology entity in the corresponding topology of the underlying SPE (who will then autonomously map it to a physical topology on computing nodes). Since this version of SPAF only accepts linear topologies, this mapping is extremely simple, being implemented as a loop visiting all nodes of the topology in an ordered way (see Figure 12).

Refactoring the Source, Processor, and Sink entities (see Figure 8), currently used to build topologies, an abstract entity TopologyNode could be created, exposing a common interface. In this way, Sources, Processors, and Sinks could be considered as TopologyNode instances, allowing to use polymorphism to implement simple (and elegant) algorithms for mapping topologies, e.g., by exploiting a *Visitor* pattern based on a topological sort of the DAG.

**Figure 13:**
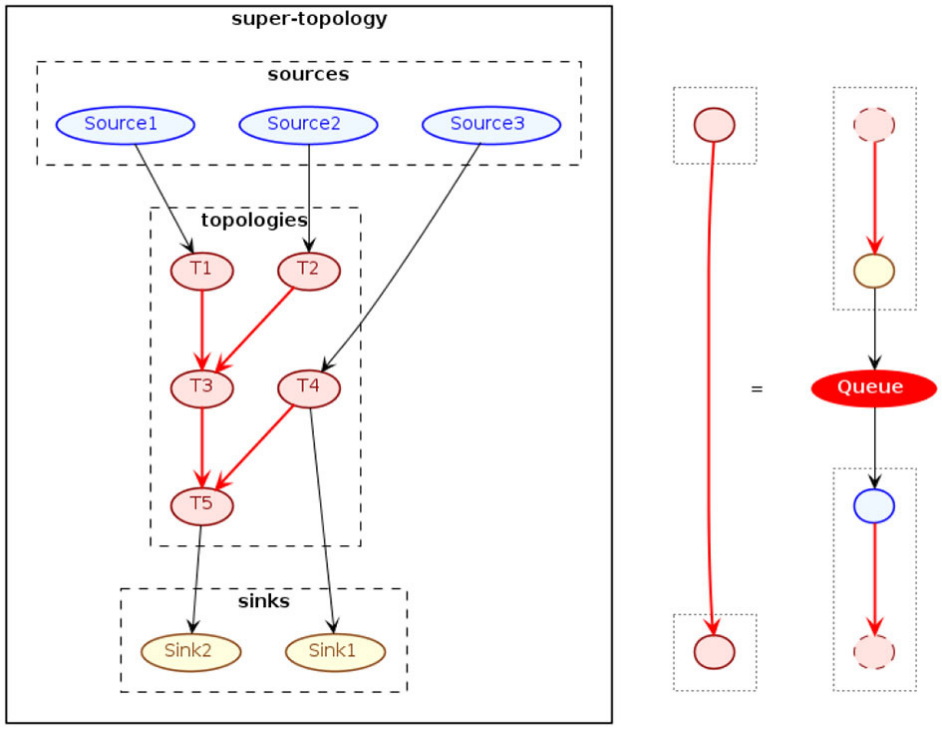
Super-topologies in SPAF.

Finally, we consider the use of multiple SPAF applications, in a simultaneous and coordinated manner, with the aim of solving a stream processing problem in a “highly distributed manner” (we will soon clarify what we mean by this adjective). The main idea is based on the decomposition of the stream processing problem into sub-problems, and in solving them through the use of multiple, independent but cooperative, SPAF projects. Each SPAF application will define a certain logical topology able of solve a certain sub-problem; each topology will receive data via the connectors provided by SPAF, process them, and send the processed data back to the outside world. In this scenario, message queues are used as means of communication between the topologies of individual projects. In other words, we can implement a topology of topologies (see Figure 13). Individual topologies, in fact, can be thought as “black boxes”, processing nodes of a DAG, receiving and sending data via the arcs connecting them, the latter realized by different message queues. What we just described corresponds precisely to the definition of topology given in Section 4.3, but at a higher level, thus the name of super-topology, where the prefix super- is to be understood in the Latin sense of “that stands above”.

In the diagram of Figure 13, it is shown how to conceptually realize a SPAF super-topology. It will be necessary to provide “border” sources and sinks (shown in blue and yellow, respectively), functioning as the input and outputs of the entire super-topology. The processing (red) nodes of the super-topology will instead correspond to a single SPAF application each, defining its own Source and Sink, and will consist of a (currently, only linear) Topology of Processors. Communication between nodes of the super-topology, i.e., between the various SPAF applications, is realized by way of appropriate message queues between node pairs (these are represented by red arcs in the figure and exemplified on the right). Obviously, each topology will have to be configured to receive and send data to the right message queues, whether they are “border” (in the example, topologies T1, T2, T4, and T5) or “internal” (T3).

The use of super-topologies in SPAF opens up some interesting scenarios:

- Each SPAF application, i.e., each node in the super-topology, can be executed by a potentially different SPE, since each application can in fact specify the desired SPAF provider independently from the others.
- It follows that each SPAF application, i.e., each node of the super-topology, can potentially be executed on a cluster of nodes by itself; for this, we previously used the term “highly distributed” execution.

The first version of the SPAF framework does not allow super-topologies to be managed at the API level. Rather, each application is independent of the others and its lifecycle must be managed by the programmer. The implementation of the illustrated scheme thus requires a systemic effort aimed at setting up the clusters of the various SPEs, the arrangement of the messaging systems, the configuration of the connectors, and the coordinated startup of the individual applications. As an interesting extension of SPAF, we are considering the implementation of facilitator tools able of automatically performing the above described tasks.

## 6. SPAF overhead

It is well known that, often, increased flexibility comes at the price of reduced performance. In this section, we investigate the impact of SPAF on performance of a MM stream analysis application.

A SPAF application is essentially composed of several layers of abstraction, which can be summarized as follows:

**Application layer:** This is the SPAF application code, defined by the programmer, which consists mainly of the code defining the Processors and the Topology.
**Interpretation layer:** It is the code of the SPAF framework, consisting of the glue-code contained in the SPAF APIs and, most importantly, the code of the mapping to the employed SPE, translating the application in terms of the underlying framework.
**Execution layer:** This is the code of the relevant underlying SPE, made usable by the SPAF provider, which actually executes the translated application.

The cost required to execute the application, both in terms of used CPU and memory, can be broken down into three components, based on the above layers.

The cost of execution of the Application Layer is totally dependent on the logic of the application. The programmer has total freedom in defining operations within Processors, and can use any third-party library. This cost is therefore difficult to predict, but it has to be paid in any case, thus the use of SPAF does not add any extra complexity.

Clearly, the cost of the Execution Layer is also independent of SPAF, and has to be paid in any case. As a matter of fact, one of the original intents of RAM3S was to help the programmer to compare performance of different SPEs in managing a stream processing application. It is obvious that using SPAF does not increase costs.

It follows that the only cost component imputable to SPAF is the one coming from the Interpretation Layer, translating the SPAF application to an application of the underlying SPE. This cost is clearly paid only once, when the SPAF application is started, and it consists in:

(1) translating the application, written with SPAF concepts, using concepts of the underlying SPE, and
(2) visiting the topology.

The first cost clearly is traded with the steepness of the learning curve, as stated in Section 4.4. If we concur that writing an application with SPAF is easier than using the original SPE, this comes at the cost of translating concepts between frameworks. The second cost is paid at runtime and can be further divided into three steps:

(1) iterating through the topology,
(2) translating each visited Processor to the underlying SPE, and
(3) executing the topology in the underlying SPE.

Steps 2 and 3 have a constant time complexity for each element, while Step 1 has a linear time complexity (this is obvious for linear topologies considered in this version of SPAF, but also with DAG topologies if we exploit a Visitor pattern as stated in Section 5). As to space complexity, since each topology component needs translation, this is again linear in the number of nodes of the topology.

## 7. Further developments

In this section, we discuss three further directions that we are considering to include in next SPAF versions: support for (1) JMS connectors, (2) configuration of the physical topology, and (3) automatic code generation through a Domain Specific Language (DSL).

### 7.1. JMS connectors

**Figure 14:**
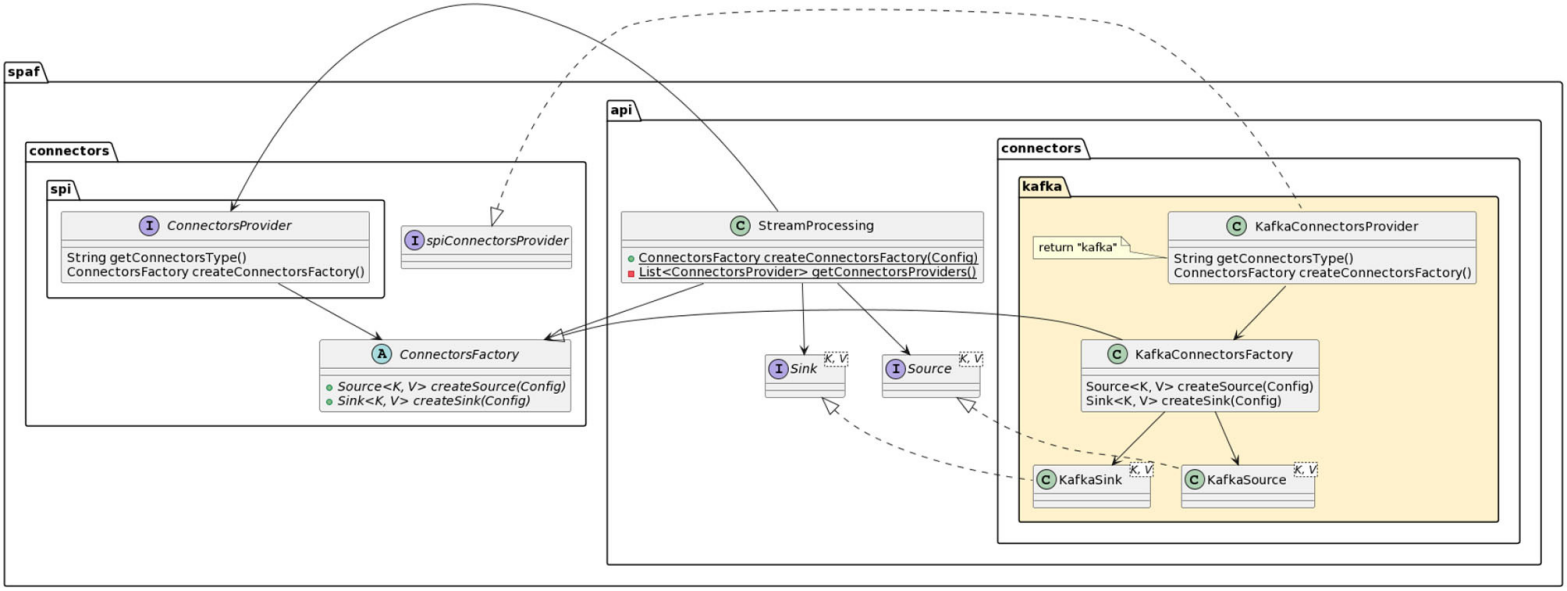
Class diagram for a connector provider descriptor (in the example, Kafka).

As we detailed in Section 3.1, RAM3S already included support for different message brokers (namely, Apache Kafka and RabbitMQ) and these have been inherited by SPAF in the Source and Sink interfaces, providing descriptors for both connector providers (see Figure 14 for details).

The difficult part in implementing a new connector in SPAF lies mainly on the provider side, since this strongly depends on the peculiarities of SPE, rather than on the message broker that one wants to connect. Implementation of a connector descriptor, on the other hand, is relatively straightforward, since it is essentially a matter of deciding which configurations have to be made available and how to represent such configurations as objects. With this in mind, it becomes apparent that the implementation of connectors for new message broker type systems can be an onerous task, thus making SPAF difficult to extend on this side. One possible way to drastically reduce the number of connectors to be realized, of both descriptive and concrete types, is to exploit the JMS [Jakarta (or Java) Messaging Service] abstraction framework.^3^ In a nutshell, JMS is an abstraction framework for using message-queue systems. To draw a parallel, we could say that SPAF stands to SPEs as JMS stands to message-queue systems. Most of the message-queue systems (including Apache Kafka and RabbitMQ) implement the JMS provider; it follows that, by realizing a single connector for JMS, SPAF would turn out to be potentially linkable to any message-queue system. If the message broker one intends to use does not include a JMS provider, it would be always possible to autonomously implement such a provider, since JMS is an open specification.

### 7.2. Physical topology configuration

A number of SPEs (like Storm and Flink) allow the programmer to control the degree of parallelism in the execution of the logical topology. This allows to determine the structure of the corresponding physical topology, in particular specifying the number of physical nodes (*tasks*) to be provided for performing the computation specified in a unique node of the logical topology (the SPAF Processor). The degree of parallelism can be specified at different levels of granularity, ranging from: system level (for all applications), individual application level (for a single Application), or individual operator (the single Processor).

**Figure 15:**
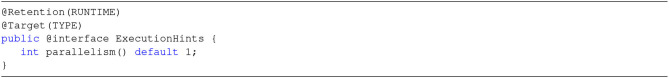
Definition of a Java annotation for specifying the degree of parallelism.

In the current version of SPAF, implemented providers do not allow to change the degree of parallelism at the level of the individual operator. SPAF APIs do not allow to specify such information, thus parallelism could only be set, in a totally arbitrary manner, for all operators. A possible future development of SPAF, which we are considering in order to allow fine tuning performance of a stream processing application at runtime, would require to expose the ability to specify the desired degree of parallelism for each Source, Sink, and Processor, at the API level. A possible means of implementation to realize this could be that of using language metadata that can be inspected at runtime, like Java Annotations (see Figure 15 for a possible example).

Such a defined annotation could be specified for each Processor as in the following, where we illustrate the case of the FaceRecognitionProcessor described in Figure 11.

 
@ExecutionHints(parallelism = 5)
public class FaceRecognitionProcessor implements
       Processor < ...> {
    @Override
    public void init() { ... }
    @Override
    public void process(...) { ... } }


### 7.3. Automating the generation of applications

In the current incarnation of SPAF, to create a new stream processing application, it is necessary to manually perform a series of tasks that are potentially repeated over different projects:

(1) writing the boilerplate code for the specific SPAF application,
(2) choosing the stream processing provider and connector provider,
(3) creating the application configuration files, and
(4) deploying the whole application on the computing infrastructure.

The open-source community has developed several tools to automate the kinds of of tasks required to prepare a new software project, like Archetypes in Maven (https://maven.apache.org/archetypes/) or Yeoman Generators (https://yeoman.io/generators/). However, these tools express their full power when they are coupled with a language used to describe the project in a declarative way. In practice, we are assuming the definition of a *Domain Specific Language* (DSL) for the high-level description of SPAF projects.

**Figure 16:**
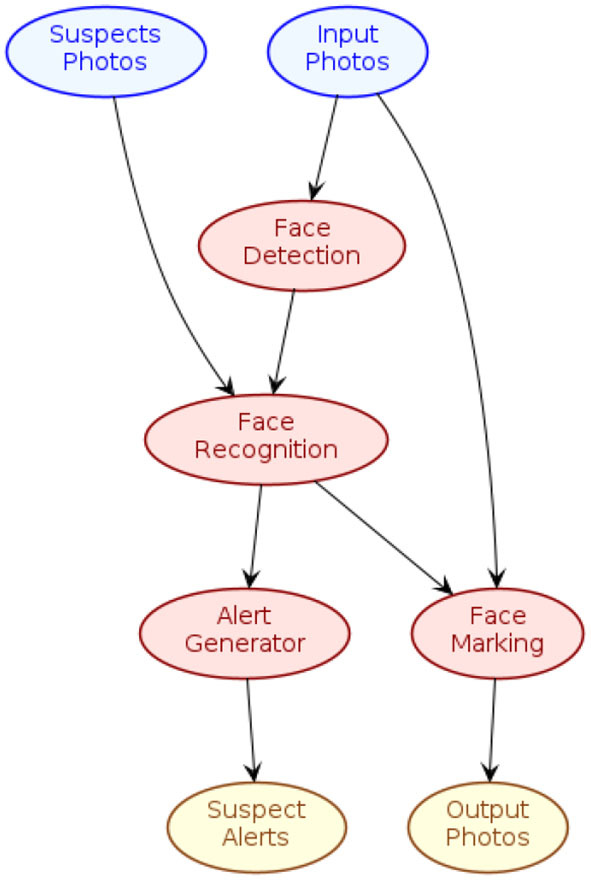
SPAF topology for an application of automatic suspect identification from videos.

To illustrate this idea, we provide an example for the use case of automatic suspect identification from videos introduced in Section 2. The topology illustrated in Figure 16 represents an evolution of the current application example for face recognition (see Section 4.4). This new topology provides the possibility of feeding, at runtime, the stream processing application with new faces to be recognized (via the “Suspects Photos” Source); such faces would be compared by the “Face Recognition” Processor with images coming from cameras (“Input Photos” Source), appropriately processed by the “Face Detection” Processor; the output of this Processor is then used by two further Processors, “Face Marking” (for drawing frames around detected faces in the original image, coloring them differently whether they were recognized or not, see Figure 2) and “Alert Generator” (which would generate a suspicious detection alert); the outputs of these two processors are then sent to two specific Sinks, “Output Photos” and “Suspect Alerts”, respectively.

By using a prototypical idea of DSL, inspired by PlantUML (https://plantuml.com/), which is the language used to define several figures in this paper, this topology could thus be described as follows:


source InputPhotos
source SuspectsPhotos
      
      
processor FaceDetection
processor FaceRecognition
processor FaceMarking
processor AlertGenerator
      
      
sink OutputPhotos
sink SuspectAlerts
      
      
InputPhotos --> FaceDetection
FaceDetection --> FaceRecognition
SuspectsPhotos --> FaceRecognition
FaceRecognition --> FaceMarking
InputPhotos --> FaceMarking
FaceMarking --> OutputPhotos
FaceRecognition --> AlertGenerator
AlertGenerator --> SuspectAlerts


An interpreter for such a language might take as input a file containing the topology description and invoke the project generator so that a more advanced boilerplate code can be produced wrt the one usually generated with the current version of SPAF (see Section 4.4). Clearly, in addition to the boilerplate code, the generator will have to produce classes to accommodate the implementation code of the Processors defined in the topology.


...
Topology topology = new Topology()
   .addSource(“InputPhotos”, inputPhotosSource)
   .addSource(“SuspectsPhotos”, suspectsPhotosSource)
   .addProcessor(“FaceDetection”, new
      FaceDetectionProcessor(), “InputPhotos”)
   .addProcessor(“FaceRecognition”, new
      FaceRecognitionProcessor(config),
      “SuspectsPhotos”, “FaceDetection”)
   .addProcessor(“FaceMarking”, new
      PersonFaceMarkingProcessor(), “InputPhotos”,      “FaceRecognition”)
   .addProcessor(“AlertGenerator”, new
      AlertGeneratorProcessor(), “FaceRecognition”)
   .addSink(“SuspectAlerts”, suspectAlertsSink,
      “AlertGenerator”)
   .addSink(“OutputPhotos”, outputPhotosSink,
      “FaceMarking”);
      
      
...


## 8. Conclusions

We introduced SPAF, a Stream Processing Abstraction Framework, as an evolution of RAM3S. As common for an abstraction framework, SPAF hides the working details of subsystems conceived for stream processing (like Samza and Storm), allowing reusability, interoperability, flexibility, and extensibility.^4^ We believe that the use of SPAF could effectively ease the development of multimedia stream data mining applications in a distributed scenario. This is clearly helpful to implement an application on top of a stream processing Big Data platform (since the developer can abstract the details specific to the framework) and also to compare performance of different stream processing platforms for a specific application, e.g., for benchmarking (Prakash, 2018). We finally highlight advantages and current limitations of SPAF by providing an analysis of its performance on a series of features (variables) that we believe are important for a software framework: These are described in Table 2, where we also provide a comparison with RAM3S, i.e., SPAF predecessor and only competitor. In this way, future versions of SPAF (and also future competitors) could be mapped to this table, highlighting their pros and cons.

**Table 2:** Comparison between RAM3S and SPAF (*n* denotes the number of nodes in the topology).

**Feature**	**RAM3S**	**SPAF**
Creation of the application	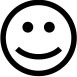 (boilerplate code, § 3.1)	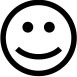 (boilerplate code, § 4.4)
Framework independence			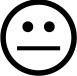 (SPE-dependent code repeated over applications, § 3.1)	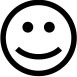 (config file, § 4.4)
Connector independence	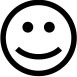 (connector-dependent code in the Receiver, § 3.1)	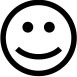 (config file, § 4.4)		
Topology shape			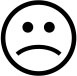 (single node topology)	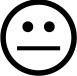 (linear topology, extensible to DAG, § 7)
Statefulness of computation	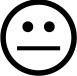 (single node topology)	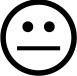 (stateless only, § 4.1)		
Physical topology			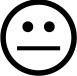 (single node topology)	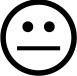 (extensible, § 7.2)
Extension to new SPEs	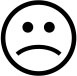 (need to write code for each app, § 4.4)	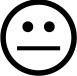 (SPE SPI, § 4)		
Extension to new connectors			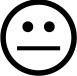 (connector-dependent code in the Receiver, § 3.1)	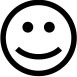 (connector SPI, § 4)
Time overhead	*O*(1) (single node topology)	*O*(*n*) (once, § 6)		
Space overhead	*O*(1) (single node topology)	*O*(*n*) (§ 6)

## Ethics statement

Written informed consent was obtained from the individual(s) for the publication of any identifiable images or data included in this article.

## Author contributions

All authors equally contributed to the conception and design of the study, the writing of the manuscript and its revision. All authors contributed to manuscript revision, read, and approved the submitted version.

## Footnotes

^1^ https://news.italy24.press/local/509606.html

^2^Other applications that have been included in RAM3S (Bartolini and Patella, 2021) are plate detection/recognition (Du et al., 2013) and printed text recognition in videos.

^3^The JMS specification is available at https://javaee.github.io/jms-spec/.

^4^Quoting David Wheeler: “All problems in computer science can be solved by another level of indirection” (Spinellis, 2007).
